# Novel Catalyst Composites of Ni- and Co-Based Nanoparticles Supported on Inorganic Oxides for Fatty Acid Hydrogenations

**DOI:** 10.3390/nano13091435

**Published:** 2023-04-22

**Authors:** Ekaterina Mamontova, Corine Trabbia, Isabelle Favier, Alejandro Serrano-Maldonado, Jean-Bernard Ledeuil, Lénaïc Madec, Montserrat Gómez, Daniel Pla

**Affiliations:** 1 Laboratoire Hétérochimie Fondamentale et Appliquée, UMR CNRS 5069, Université Toulouse III—Paul Sabatier, 118 Route de Narbonne, CEDEX 9, 31062 Toulouse, France; ekaterina.mamontova@umontpellier.fr (E.M.); corine.trabbia@gmail.com (C.T.); isabelle.favier@univ-tlse3.fr (I.F.); alejandro.serrano@univ-tlse3.fr (A.S.-M.); 2 E2S UPPA, CNRS, IPREM, Université de Pau et des Pays de l’Adour, 64053 Pau, France; jledeuil@univ-pau.fr (J.-B.L.); lenaic.madec@univ-pau.fr (L.M.)

**Keywords:** nickel-based nanoparticles, magnesium aluminum oxide spinel, titanium dioxide anatase, C=C bonds hydrogenation, fatty acids, valorization, industrial waste

## Abstract

In the quest to develop nanometrically defined catalytic systems for applications in the catalytic valorization of agri-food wastes, small Ni-based nanoparticles supported on inorganic solid supports have been prepared by decomposition of organometallic precursors in refluxing ethanol under H_2_ atmosphere, in the presence of supports exhibiting insulating or semi-conductor properties, such as MgAl_2_O_4_ and TiO_2_, respectively. The efficiency of the as-prepared Ni-based nanocomposites has been evaluated towards the hydrogenation of unsaturated fatty acids under solvent-free conditions, with high selectivity regarding the hydrogenation of C=C bonds. The influence of the support on the catalytic performance of the prepared Ni-based nanocomposites is particularly highlighted.

## 1. Introduction

Despite the fact that the valorization of fatty wastes in the form of fatty esters has long been studied, the corrosive nature of fatty acids makes their transformation into free acid form a more challenging quest in terms of scaling up or intensification for industrial applications provided that robust catalyst solutions withstanding the reaction conditions are found. Since the seminal works of Paul Sabatier regarding nickel reactivity [1,2] and its further applications in catalytic hydrogenations [3,4], the quest for attaining high activity and selectivity while overriding deleterious catalyst passivation pathways has resulted in a number of preparations, with the aim of preserving the catalyst dispersion due to its key implications towards reactivity [5]. Raney nickel was described for the first time in 1925 [6] and Raney cobalt in 1933 [7]; intermetallic nickel–aluminum and cobalt–aluminum alloys, respectively, featuring mesoporous structures upon activation via alkaline treatment, have been widely used as hydrogenation catalysts [5,8], such as for the industrial production of hexamethylenediamine from adiponitrile [9] and margarine both from vegetable oils and animal fats [10]. In particular, the correlation between Raney nickel catalyst microstructure (NiAl_3_ vs. Ni_2_Al_3_) and catalytic performance has been recently established [11], providing new means towards rational catalyst design involving inorganic supports. Later on, Ni-based Urushibara [12] and Kieselguhr hydrogenation catalysts [13,14], as well as cobalt-based ones [15,16,17], have also been reported in the literature, affording new means to attain zero-valent nickel and cobalt phases that play essential roles in catalytic C=C bond hydrogenation reactions. To reduce Ni soap formation, fatty acid hydrogenations are generally carried out at high pressures (20–30 bar) and temperatures in the range of 180–210 °C with pre-dried samples [18].

A particular type of composite materials made of Al_2_O_3_–Ni and Al_2_O_3_–NiO with a spinel-phase NiAl_2_O_4_ [19] have been used as (pre)catalysts for reforming reactions due to their high thermal stability [20,21]. Many preparative protocols under a sintering atmosphere [13,14,15,16] have been described (namely, powder coating [22], sol–gel [23], hydrothermal and solvothermal methods [10], and impregnation [12]), triggering in some cases the structural transformation of NiAl_2_O_4_ from normal spinel (Ni^2+^)[Al_2_^3+^]O_4_ to an inverse one, (Al^3+^)[Ni^2+^Al^3+^]O_4_ [24]. The corrosion constraints associated with the hydrogenation of fatty acids and potential catalyst deactivation pathways poised us to select neutral metal oxide supports with enhanced thermal stability and mechanical robustness, such as titanium dioxide (TiO_2_) and magnesium aluminum oxide spinel (MgAl_2_O_4_) owing to their relatively high activity in hydrogenations and reforming processes [25,26,27,28], low cost, and wide availability.

Nanocatalysis provides innovative solutions towards catalyst enhancements via kinetic stabilization at the nanoscale of small particles, thus providing high surface-metal ratios together with a number of highly reactive metal sites arising from low-coordination defects, as well synergistic effects at the metal-support interfaces for heterogeneized systems [29]. Based on our previous experience [30,31], herein we describe the synthesis of original mono-metallic and bi-metallic Ni and/or Co-based nanocomposites by a one-step methodology, resulting in well-defined metal nanoparticles (MNPs) supported on TiO_2_ or MgAl_2_O_4_. Working under smooth conditions, this approach permits a straightforward MNPs incorporation on the support surface, as well as overriding potential structural changes of the supports. These as-prepared catalytic materials exhibited remarkable activity in the hydrogenation processes of fatty acids, including their application in waste valorization.

## 2. Materials and Methods

### 2.1. Materials

All reagents were purchased at the highest commercial quality and used without further purification unless stated otherwise. Halloysite and quinidine were dried in a Schlenk flask under vacuum at 100 °C overnight prior to use. The synthesis of metal nanoparticles was performed in Fisher–Porter bottles. The synthesized catalytic materials were isolated by centrifugation and dried under vacuum at 100 °C overnight; the dried materials were stored in a glove box. Solvents were previously dried on a solvent purifier system and degassed via three freeze-pump-thaw cycles. High-pressure hydrogenation reactions were carried out in stainless steel autoclaves acquired from Parr and Top Industries.

For general experimentation details and characterization techniques, see Supplementary Materials.

### 2.2. Synthesis of Supported Metal Nanoparticles

#### 2.2.1. Synthesis of Nickel Nanoparticles Stabilized by Quinidine and Supported on MgAl_2_O_4_ or TiO_2_, **NiNP@MgAl_2_O_4_**, and **NiNP@TiO_2_**, Respectively

A Fisher–Porter bottle was charged with bis(1,5-cyclooctadiene)nickel(0) (234.3 mg, 0.85 mmol), quinidine (276.4 mg, 0.85 mmol), and the support (1 g of MgAl_2_O_4_ or TiO_2_-P90) and then sealed with a septum inside the glove box. The Fisher–Porter bottle was then removed from the glove box, and the solid mixture was suspended in degassed EtOH (32 mL) under an argon atmosphere prior to sealing the Fisher–Porter with its head. The system was pressurized with H_2_ (3 bar) at room temperature and then heated up to 100 °C and stirred for 18 h. A black dispersion was obtained and transferred to a centrifuge tube via cannulation under Ar. Centrifugation was carried out at 3500 rpm for 10 min. After removing the supernatant, the solid was dispersed in degassed EtOH (rinsing repeated three times). The obtained black solid was dried under a vacuum overnight and stored in the glove box prior to use. **NiNP@MgAl_2_O_4_**: 750 mg (72% yield); **NiNP@TiO_2_**: 1000 mg (95% yield).

#### 2.2.2. Synthesis of Cobalt Nanoparticles Stabilized by Quinidine and Supported on MgAl_2_O_4_ and TiO_2_, **CoNP@MgAl_2_O_4_**, and **CoNP@TiO_2_**

A Fisher–Porter bottle was charged with dicobalt octacarbonyl (145.1 mg, 0.42 mmol), quinidine (275.1 mg, 0.85 mmol), and the support (1 g of MgAl_2_O_4_ or TiO_2_-P90) and then sealed with a septum inside the glove box. The Fisher–Porter bottle was then removed from the glove box, and the solids were suspended in degassed EtOH (32 mL) under Ar prior to sealing the Fisher–Porter with its head. The system was pressurized with H_2_ (3 bar) at room temperature and then heated to 100 °C and stirred for 18 h. A black dispersion was obtained and transferred to a centrifuge tube via cannulation under Ar. Centrifugation was carried out at 3500 rpm for 10 min. After the removal of the supernatant, the solid was dispersed in degassed EtOH (rinsing repeated three times). The obtained black solid was dried under a vacuum overnight and stored in the glove box prior to use. **CoNP@MgAl_2_O_4_**: 570 mg (54% yield); **CoNP@TiO_2_**: 993 mg (94% yield).

#### 2.2.3. Synthesis of Nickel–Cobalt Nanoparticles Stabilized by Quinidine and Supported on MgAl_2_O_4_ or TiO_2,_ NiCoNP@MgAl_2_O_4_, and NiCoNP@TiO_2_, Respectively

A Fisher–Porter bottle was charged with dicobalt octacarbonyl (36.9 mg, 0.11 mmol), bis(1,5-cyclooctadiene)nickel(0) (59.1 mg, 0.22 mmol), quinidine (139.4 mg, 0.42 mmol), and the support (500 mg of MgAl_2_O_4_ or TiO_2_-P90) and then sealed with a septum inside the glove box. The Fisher–Porter bottle was then removed from the glove box, and the solid mixture was suspended in degassed EtOH (16 mL) under Ar prior to sealing the Fisher–Porter with its head. The system was pressurized with H_2_ (3 bar) at room temperature and then heated to 100 °C and stirred for 18 h. A black dispersion was obtained and transferred to a centrifuge tube via cannulation under Ar. Centrifugation was carried out at 3500 rpm for 10 min. After the removal of the supernatant, the solid was dispersed in degassed EtOH (rinsing repeated three times). The obtained black solid was dried under a vacuum overnight and stored in the glove box prior to use. **NiCoNP@MgAl_2_O_4_**: 450 mg (84% yield); NiCoNP@TiO_2_: 520 mg (97% yield).

#### 2.2.4. Extraction of Metal Nanoparticles from the Inorganic Support with Glycerol

For characterization purposes, 70 mg of catalytic material was dispersed in 5 mL of glycerol. The mixture was stirred overnight at room temperature. A black dispersion was obtained and transferred to a centrifuge tube via cannulation under Ar. Centrifugation was carried out at 3500 rpm for 15 min. The obtained glycerol phase was analyzed by TEM to determine average nanoparticle diameters with size distributions.

### 2.3. Catalytic Hydrogenation Reactions Using **NiNP@MgAl_2_O_4_**

A small glass flask containing 12 mg (0.01 mmol Ni) of nickel nanoparticles **NiNP@MgAl_2_O_4_** with 285 mg of oleic acid (1 mmol), weighted in a glove box, was introduced in an autoclave under an argon atmosphere. The mixture was then pressurized under 5 bar H_2_ and heated at 250 °C with an aluminum heating block for 30 min. At the end of the reaction, the organic products were extracted with dichloromethane (5 × 3 mL), the solution was filtered through a 0.6-μm PTFE syringe filter, and the solvent was evaporated under a vacuum. Conversion and selectivity were determined by ^1^H NMR using 4-methylanisole as the internal standard. Reported catalytic results correspond to the mean values obtained for three replicates.

## 3. Results and Discussion

### 3.1. Design and Characterization of Nanocomposite Materials

**Figure 1:**
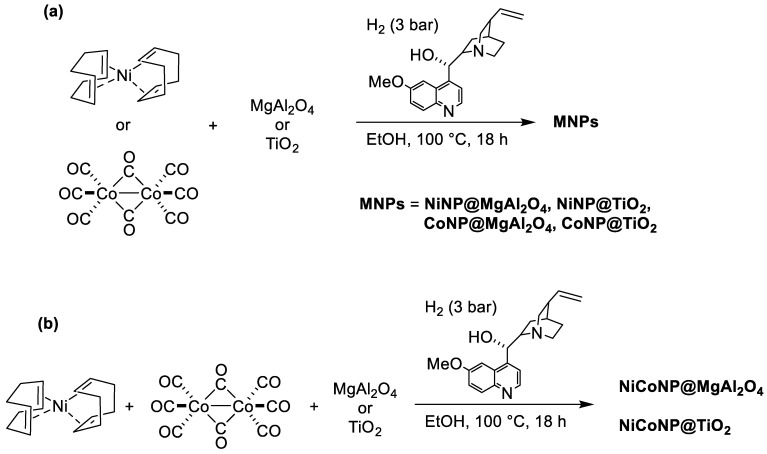
(**a**) Synthesis of both mono-metallic Ni and Co nanocomposites supported on MgAl_2_O_4_ or TiO_2_; (**b**) synthesis of bi-metallic NiCo nanocomposites supported on MgAl_2_O_4_ or TiO_2_.

With the aim of efficiently immobilizing mono-metallic Ni- (NiNP), Co- (CoNP), and bi-metallic NiCo nanoparticles (NiCoNP) on inorganic oxides (MgAl_2_O_4_ and TiO_2_), we used herein a methodology based on the decomposition of organometallic precursors, either bis(1,5-cyclooctadiene)nickel(0), octacarbonyldicobalt(0), or a combination of both under H_2_ atmosphere (3 bar) in the presence of quinidine as a stabilizer and a suspension of the solid support in ethanol under stirring at 100 °C overnight, adapting a methodology that has been recently described by our group for the preparation of Ni-based halloysite nanocomposites [31]. Thus, the synthesis of six nanocomposite materials, namely **NiNP@MgAl_2_O_4_**, **CoNP@MgAl_2_O_4_**, **NiCoNP@MgAl_2_O_4_**, **NiNP@TiO_2_**, **CoNP@TiO_2_**, and **NiCoNP@TiO_2_** was achieved (Figure 1). For the bi-metallic systems, a nickel:cobalt ratio of 1:1 was used.

**Figure 2:**
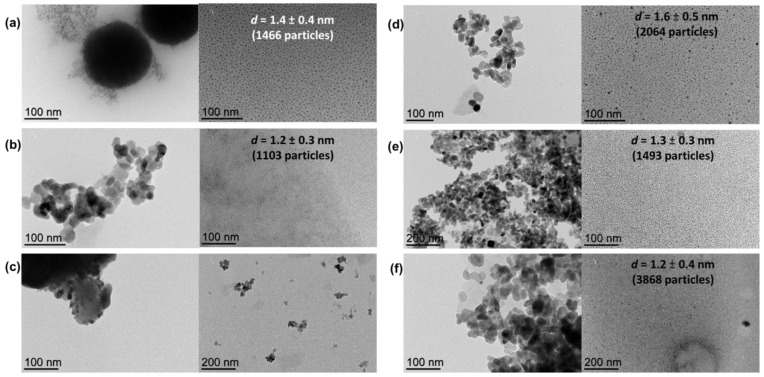
TEM micrographs of nickel-based nanocomposite materials: (**a**) **NiNP@MgAl_2_O_4_** after synthesis and glycerol extracts with particle size distribution (1.4 ± 0.4 nm for 1466 particles); (**b**) **CoNP@MgAl_2_O_4_** after synthesis and glycerol extracts with particle size distribution (1.2 ± 0.3 nm for 1103 particles); (**c**) **NiCoNP@MgAl_2_O_4_** after synthesis and glycerol extracts; (**d**) **NiNP@TiO_2_** after synthesis and glycerol extracts with particle size distribution (1.6 ± 0.5 nm for 2046 particles); (**e**) **CoNP@TiO_2_** after synthesis and glycerol extracts with particle size distribution (1.3 ± 0.3 nm for 1493 particles); (**f**) and **NiCoNP@TiO_2_** after synthesis and glycerol extracts with particle size distribution (1.2 ± 0.4 nm for 3868 particles) (see Figures S1–S6 for further TEM micrographs of each material in the Supplementary Materials).

The sizes of metal NPs could not be directly estimated by TEM from the as-prepared composites due to the difficulty of distinguishing the metal NPs from the support; therefore, metal NPs were extracted from the corresponding support with glycerol for characterization purposes taking advantage of the high affinity of MNPs for the glycerol phase [32,33,34,35]. As observed by TEM analyses of the glycerol dispersions (direct analyses thanks to the negligible vapor pressure of glycerol), well-dispersed small Ni nanoparticles were obtained from both MgAl_2_O_4_ and TiO_2_ supports (mean diameter: 1.4 ± 0.4 nm and 1.6 ± 0.5 nm for **NiNP@MgAl_2_O_4_** and **NiNP@TiO_2_**, respectively, for 1466 and 2064 particles, respectively; Figure 2), in agreement with our previous contributions involving nickel nanoparticles [30,31]. For the analogous mono-metallic Co systems, well-dispersed small Co nanoparticles were also obtained independently from the support used (mean diameter: 1.2 ± 0.3 nm and 1.3 ± 0.3 nm for **CoNP@MgAl_2_O_4_** and **CoNP@TiO_2_**, respectively, for 1103 and 1493 particles, respectively). Moreover, for the NiCo bi-metallic systems, the glycerol extraction was only efficient for **NiCoNP@TiO_2_** (mean diameter: 1.2 ± 0.4 nm for 3868 particles).

**Figure 3:**
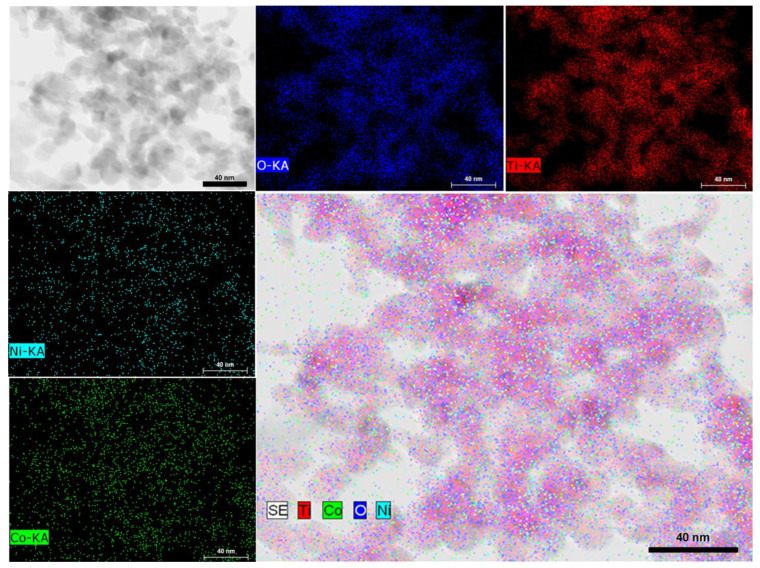
STEM-BF analysis of **NiCoNP@TiO_2_** showing elemental distribution of Ti (red), Co (green), Ni (cyan), and O (blue).

Scanning Transmission Electron Microscopy Bright Field (STEM-BF), mapping on an HRTEM image corresponding to **NiCoNP@TiO_2_** evidenced a homogeneous dispersion of nickel and cobalt over the support surface, suggesting an alloy structure (Figure 3 and Figure S7 in the Supplementary Materials for another HRTEM image). However, the small size of the as-prepared nanoparticles lies at the limit of current characterization techniques. It is worth mentioning that the insulating nature of MgAl_2_O_4_ as support hampered HRTEM analysis due to charging effects observed during acquisition at 200 KV.

ICP-AES analyses of the as-prepared materials were consistent with an efficient metal deposition over both supports: 4.6 wt% Ni for both **NiNP@MgAl_2_O_4_** and **NiNP@TiO_2_**; 4.5 wt% Co for both **CoNP@MgAl_2_O_4_** and **CoNP@TiO_2_**; 3.0 wt% Ni and 1.8 wt% Co for **NiCoNP@MgAl_2_O_4_**, as well as 2.8 wt% Ni and 2.6 wt% Co for **NiCoNP@TiO_2_** (expected data: 5 wt% Ni, 5 wt% Co or overall Ni–Co metal content). In addition, the presence of C and N in the as-prepared materials could be determined by elemental analyses, evidencing the presence of quinidine acting as a stabilizer in the final materials (N content: lower than 0.3 wt% for all composite materials, see Table S1 in the Supplementary Materials for further details). Powder X-ray diffraction analyses of the four nanocomposites only exhibited the corresponding peaks of MgAl_2_O_4_ and TiO_2_ supports; probably, the 5 wt% metal loading (of Ni and/or Co) content falling below the limits of detection for this technique, together with potential peak broadening effects arising from the small size of NiNP and NiCoNP, in particular for **NiNP@MgAl_2_O_4_**, **NiNP@TiO_2_**, and **NiCoNP@TiO_2_** (see Figures S8–S13 in the Supplementary Materials).

With the aim of assessing the presence of quinidine or potential metal oxides on the as-prepared Ni-based materials, diffuse reflectance infrared Fourier transform spectroscopy (DRIFTS) analyses were carried out (Figures S14 and S15 in the Supplementary Materials). For **NiNP@TiO_2_**, **CoNP@TiO_2_ NiCoNP@TiO_2_** absorption bands corresponding to C–H stretching of quinidine (ca. 3000–2840 cm^−1^) were identified together with the characteristic bands in the region ca. 1630–1450 cm^−1^, confirming the presence of quinidine (Figure S14 in the Supplementary Materials). In agreement with the lower quinidine content determined by elemental analysis for MgAl_2_O_4_ spinel composites, the qualitative presence of quinidine in **NiNP@MgAl_2_O_4_**, **CoNP@MgAl_2_O_4_**, and **NiCoNP@MgAl_2_O_4_** could not be assessed by DRIFTS (see Figure S15 in the Supplementary Materials). In addition, the intense absorption bands obtained for these composites in the region of 900–450 cm^−1^ corresponding to Al–O and Mg–O bond stretching vibrations of the support, do not permit to exclude the presence of metal oxides (stretching vibrations: 700–600 cm^−1^ for Ni–O and 680–558 cm^−1^ for Co–O)see Figure S15 in the Supplementary Materials).

**Figure 4:**
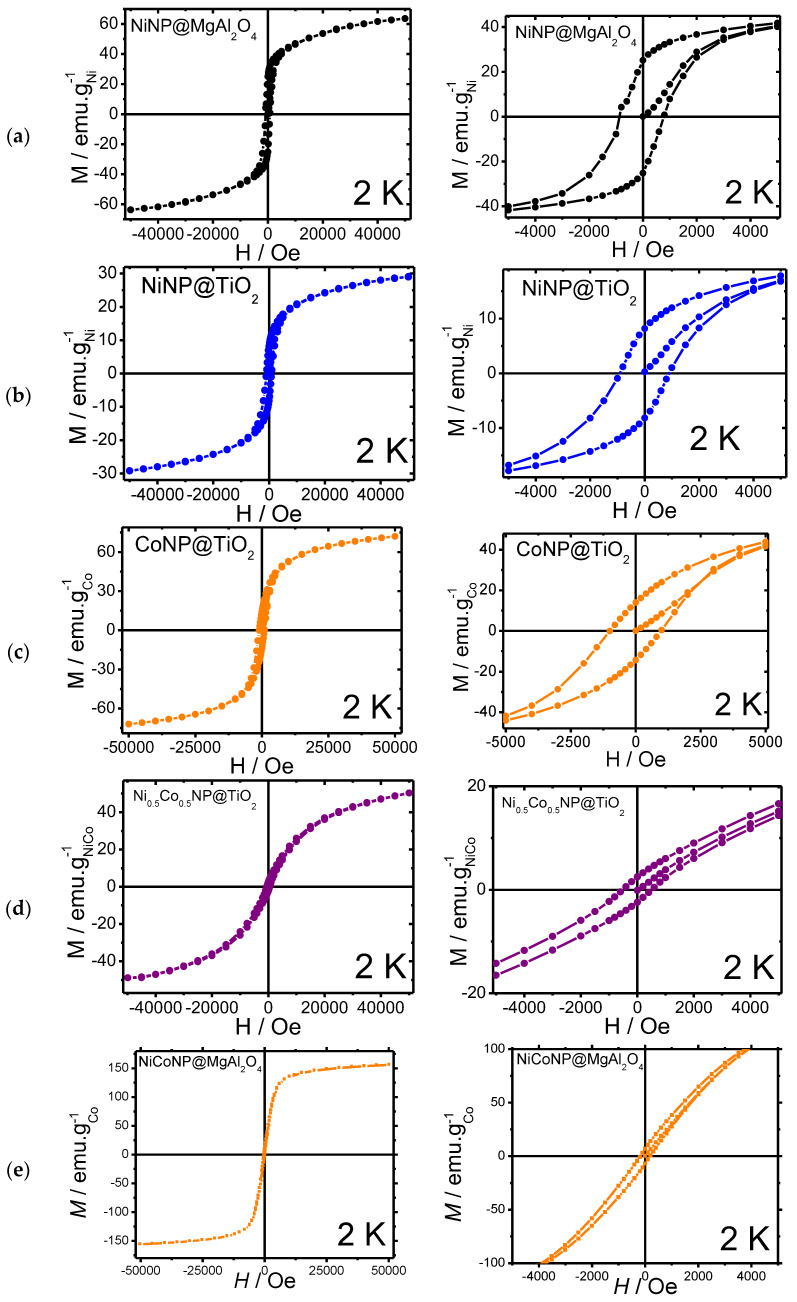
Magnetization curves with magnification: (**a**) **NiNP@MgAl_2_O_4_**; (**b**) **NiNP@TiO_2_**; (**c**) **CoNP@TiO_2_**; (**d**) **NiCoNP@TiO_2_**; (**e**) **NiCoNP@MgAl_2_O_4_**.

Moreover, magnetic measurements were carried out for the Ni-based nanocomposites at 2 K (Figure 4, Table 1). Magnetic measurements for the mono-metallic **NiNP@MgAl_2_O_4_** and **NiNP@TiO_2_** systems were carried out, showing a weak ferromagnetic behavior for both materials, with a narrow hysteresis at 2 K with coercive fields of 775 and 947 Oe, respectively, together with moderate remanence magnetization (25.2 and 8.3 emu/gNi, respectively). The saturation magnetization values (M_s_) for **NiNP@MgAl_2_O_4_** (63.7 emu/g) were similar to those reported for bulk nickel (54 emu/g) [36,37]; however, for **NiNP@TiO_2_** the M_s_ (29 emu/g) was lower probably due to either spin capping effects from stabilizers over the surface of nickel nanoparticles or the presence of nickel oxides.

In comparison to the mono-metallic composites, magnetic measurements of the bi-metallic systems **NiCoNP@MgAl_2_O_4_** and **NiCoNP@TiO_2_** showed a superparamagnetic behavior, with narrow hystereses at 2 K and coercive field values of 511 and 181 Oe, respectively, together with the lowest remanence magnetizations (6.7 and 2.7 emu/gNiCo, respectively). Moreover, **NiCoNP@MgAl_2_O_4_** and **NiCoNP@TiO_2_** presented lower M_s_ values (156.2 and 52.0 emu/gNiCo) than the value for Co-hcp (162 emu/g) [38]. The lower value obtained for **NiCoNP@TiO_2_** in comparison to the Ni reference (54 emu/g for bulk Ni) could be due to the presence of nickel and cobalt oxides or the presence of capping agents, such as quinidine [39,40,41,42].

Given the fast oxidation of the samples during the introduction to the experimental station, X-ray absorption spectroscopy (XAS) analyses of **NiNP@MgAl_2_O_4_**, **NiNP@TiO_2_**, **NiCoNP@MgAl_2_O_4_**, and **NiCoNP@TiO_2_** could only confirm the presence of nickel and/or cobalt oxides, mainly attributed to NiO or Ni(OH)_2_ (Figure S16 in the Supplementary Materials) and CoO (Figure S17 in the Supplementary Materials) [43].

**Figure 5:**
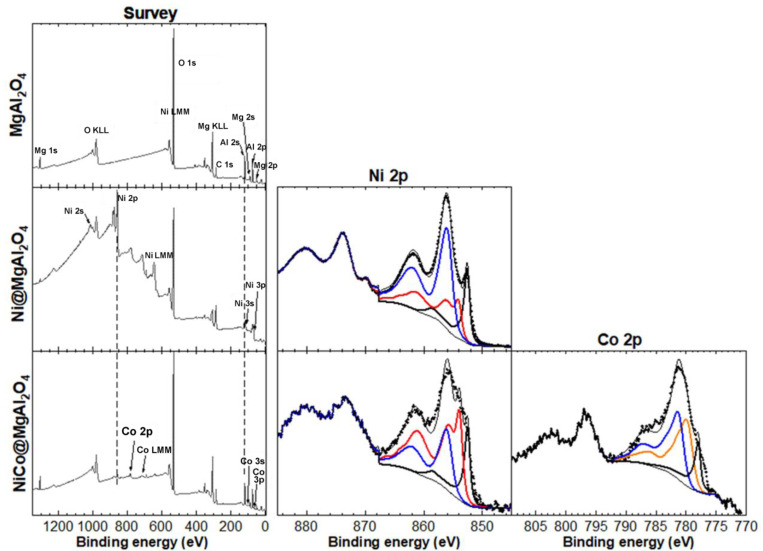
XPS analyses: XPS survey spectra of MgAl_2_O_4_ support, **NiNP@MgAl**_2_**O**_4_ and **NiCoNP@MgAl**_2_**O**_4_ composites (left column); high-resolution spectrum at the binding energy region of Ni 2p; black, red, and blue continuous traces correspond to Ni(0), NiO, and Co(OH)_2_ envelopes used to fit the experimental data (dotted line); the fit was carried out on the Ni 2p_3/2_ binding energy (middle column); high-resolution spectrum at the binding region of Co 2p; black, orange, and blue continuous traces correspond to Co(0), CoO, and Co(OH)_2_ envelopes used to fit the experimental data (dotted line); the fit was carried out on the Co 2p_3/2_ binding energy (right column). For the peak fitting procedures, see the experimental section.

Considering the high catalytic performance of both **NiNP@MgAl_2_O_4_** and **NiCoNP@MgAl_2_O_4_** (see below, Section 3.2), further characterization of these composite materials was performed. X-Ray photoelectron spectroscopy (XPS) analyses were carried out to determine the oxidation state of nickel and cobalt species immobilized on MgAl_2_O_4_, as well as the elements present at the surface of the catalytic materials (Figure 5). The XPS survey spectrum of the MgAl_2_O_4_ support showed the expected peaks for Mg, Al, and O. The XPS quantification showed that MgAl_2_O_4_ accounts for about 24 at.% while Al_2_O_3_ is also present with about 60 at.% in accordance to previous reports concerning solid solutions of Al_2_O_3_ phases in MgAl_2_O_4_ closer to the composition of stoichiometric spinel [44]. For **NiNP@MgAl_2_O_4_**, peaks for Ni were also observed, with a Ni content of 13 at.%. Notably, the Ni 2p XPS core level spectra showed three contributions from Ni metal (18%), NiO (26%), and Ni(OH)_2_ (56%). For **NiCo@MgAl_2_O_4_**, peaks for both Ni and Co were also observed, which account for a low content of about 1 at.% each. Significantly, the Ni 2p XPS core level spectra showed three contributions from Ni metal (17%), NiO (53%), and Ni(OH)_2_ (50%), and the Co 2p XPS core level spectra showed three contributions from Co metal (16%), CoO (40%), and Co(OH)_2_ (44%) (see Figure S18 and Table S2 in the Supplementary Materials). Despite the fact that Co_3_O_4_ is thermodynamically more stable than CoO, the coexistence of Co(II) and Co(III) species is difficult to assess given their close binding energies (779.5 and 781.3 eV, respectively) [39,40,41,42,45]. However, a better fitting of the Co 2p_3/2_ XPS core level spectra with the envelopes corresponding to Co 2p_3/2_ peaks of the following references Co(0), Co_3_O_4_, CoO, Co(OH)_2_ was obtained with a slight constraint on the position of each, where the presence of Co_3_O_4_ fell to zero. It is noteworthy to highlight that although Co_3_O_4_ could only be estimated if CoO was imposed to be zero, Co(0) content was similar for both fittings. Overall, these results highlight that the preparation method for **Ni@MgAl_2_O**_4_ and **NiCo@MgAl_2_O_4_** is effective in obtaining mono and bi-metallic NPs, albeit with partial oxidation. It is important to mention that due to the rapid oxidation of Ni(0) and Co(0), even under an Ar glove box atmosphere or ultra-high vacuum, XPS analyses of such powder samples were a challenge. Consequently, the real at.% of Ni and Co metal in the samples is probably higher than the values reported herein.

### 3.2. Catalytic Hydrogenation of Fatty Acids

The catalytic behavior of the six as-prepared nanocomposites was assessed in the hydrogenation of oleic acid (**1**) towards stearic acid (**2**) as a benchmark reaction (Table 2). Under neat conditions, working at 180 °C, 5 bar H_2_ pressure, and 1 mol% of metal loading (for the bi-metallic catalyst, 0.5 mol% Ni and 0.5 mol% Co), titania nanocomposites **CoNP@TiO_2_**, **NiNP@TiO_2_**, and **NiCoNP@TiO_2_** only gave low to moderate conversions (10%, 65%, and 31% conversions, respectively; entries 1–3, Table 2), highlighting the positive impact of mono-metallic NiNPs in terms of catalyst efficiency (entry 2 vs. 1 and 3, Table 2). In agreement with this trend, the analogous MgAl_2_O_4_ spinel-based nanocomposite system with CoNPs, **CoNP@MgAl_2_O_4_**, also exhibited a poor performance (12% conversion; entry 4, Table 2). Nevertheless, **NiNP@MgAl_2_O_4_** and **NiCoNP@MgAl_2_O_4_** were more active than titania-derived catalytic materials (96 and 93% conversions, respectively; entries 5–6 vs. 2–3, Table 2), exhibiting both comparable efficiencies at 1 mol% metal catalyst loading (entries 5–6, Table 2). Thus, both mono-metallic and bi-metallic MgAl_2_O_4_-based composites displayed a better performance than the corresponding TiO_2_ counterparts.

Despite the lower conversions obtained for **NiCoNP@TiO_2_** and **NiCoNP@MgAl_2_O_4_** in comparison to the mono-metallic nickel counterparts at 1 mol% catalyst loadings (entries 3 and 6 versus entries 2 and 5, Table 2), the poorest performance of Co could be concluded at this catalyst loading. However, it is worth mentioning that Co is permitted to operate at sub-mol% catalyst loadings with **NiCoNP@MgAl_2_O_4_**, probably hampering the deactivation of the Ni phase. Whereas the presence of oxidized nickel species such as NiO can be attributed to lower catalyst efficiency towards C=C hydrogenation of fatty acids as they usually require a preactivation step to, in situ, generate catalytically active Ni(0) species [46], cobalt oxides are well-known catalysts promoting the reduction of carboxylic acid function as well as hydrodeoxygenation of the corresponding fatty alcohols, albeit under harsher conditions (H_2_ pressure and temperature) [47].

Despite the reasonably better performance of the bi-metallic composite **NiCoNP@MgAl_2_O_4_** than **NiNP@MgAl_2_O_4_** (entries 7–8, Table 2), we decided to pursue the reaction scope studies with the mono-metallic catalyst due to its better activity at 1 mol% Ni loading and easier characterization. It is worth mentioning that no significant loss of fatty acids by adsorption on any of the six catalytic composites was observed, recovering quantitatively the organic compounds. For further optimization parameters, see Table S3 in the Supplementary Materials.

Substrate scope studies using other mono- and poly-unsaturated C_18_ fatty acids were then performed (Table 3). Elaidic acid, the (*E*)-isomer of oleic acid, was efficiently hydrogenated albeit in lower conversion (72% conversion; entry 1, Table 3) in comparison to oleic acid (96% conversion; entry 5, Table 3). Linoleic acid (**4**), showing two C=C bonds at C_9_ and C_12_, led to 69% of mono-unsaturated acid and 31% of stearic acid with nearly full conversion (entry 2, Table 3). Moreover, α-linolenic acid (**5**), featuring three C=C bonds at C_9_, C_12_, and C_15_ positions, only gave 86% conversion to mono-unsaturated fatty acids (entry 3, Table 3); longer reaction time (4 h) and higher catalyst loading (2 mol% Ni) were required to completely transform both linoleic and linolenic acids to the fully saturated stearic acid (entries 4–5, Table 3). The determination of metal traces present in the organic extracts after the 4 h benchmark reaction using **NiNP@MgAl_2_O_4_** catalyst revealed the presence of trace amounts of Ni (1.1 ppm by ICP-AES close to the detection limit of this technique), revealing negligible leaching of the catalyst and pointing to a surface reactivity of the catalyst.

From an application viewpoint, we assessed the efficiency of **NiNP@MgAl_2_O_4_** towards the valorization of fatty wastes coming from agri-food industries via selective C=C double-bond hydrogenation; the fully saturated products, namely stearic and palmitic acids and mixtures thereof are found in a large variety of products (e.g., food supplements, emulsifiers, surfactants, cosmetics, and plastics) [48]. In particular, a sample from duck fat waste supplied by the enterprise SAPOVAL was used for this study. Thus, **NiNP@MgAl_2_O_4_** showed high efficiency in the hydrogenation of the agri-food waste, mainly constituted 76% of unsaturated fatty acids and 24% of saturated ones (stearic acid, 20%; palmitic acid, 4%). At 180 °C for 4 h using 2 mol% Ni under 5 bar H_2_, the hydrogenation of the agri-food waste took place quantitatively, giving a highly enriched stearic acid sample (entry 6, Table 3).

## 4. Conclusions

In this contribution, a general strategy encompassing the preparation of first-row nanocomposites based on Co and Ni nanoparticles supported on MgAl_2_O_4_ spinel or TiO_2_ and their use as sustainable catalytic materials towards selective hydrogenation processes of fatty acid substrates is reported. Thus, six original and well-defined mono-metallic (**NiNP@MgAl_2_O_4_**, **CoNP@MgAl_2_O_4_**, **NiNP@TiO_2_**, and **CoNP@TiO_2_**) and bi-metallic (**NiCoNP@MgAl_2_O_4_** and **NiCoNP@TiO_2_**) nanocomposite materials were prepared by one-step procedure following an organometallic bottom-up approach under smooth conditions, and fully characterized by (HR)TEM, ICP, PXRD, FTIR, XPS, XAS, and magnetization. The catalytic performance of MgAl_2_O_4_ and TiO_2_–based composites towards fatty acid hydrogenation was evaluated, revealing the superior role of the former as support for the immobilization of mono- and bi-metallic Ni and Co nanoparticles, where the choice of MgAl_2_O_4_ support presenting both acidic and basic sites might play a better role than the amphoteric properties of TiO_2_, which can induce catalyst deactivation such as the formation of Ni soaps known to block the active catalyst surface [18].

Despite their prone oxidation, XPS analysis was instrumental in assessing the content of zero-valent nickel and cobalt in the as-prepared nanocomposites. Their efficacy towards the C=C bond hydrogenation of fatty acids was evaluated. The two nickel-based systems supported on MgAl_2_O_4_, namely the mono-metallic **NiNP@MgAl_2_O_4_** and the bi-metallic **NiNP@MgAl_2_O_4_**, showed the best catalytic performances operating at 1 mol% catalyst loading and 180 °C for 30 min under 5 bar H_2_ pressure. Despite the slightly lower conversion obtained for **NiCoNP@MgAl_2_O_4_** in comparison to the mono-metallic **NiNP@MgAl_2_O_4_** at 1 mol% catalyst loadings (93 and 96% conv., respectively), this bi-metallic composite permitted to work at sub-mol% catalyst loadings (0.6 mol%) maintaining a good conversion and exclusive selectivity towards stearic acid. **NiNP@MgAl_2_O_4_** was successfully applied in the selective C=C bond hydrogenation of mono- and poly-unsaturated C_18_ fatty acids, including waste from agri-food industrial residues. Overall, the **NiNP@MgAl_2_O_4_** catalyst offers great promise to carry out free fatty acid hydrogenations under milder H_2_ pressures (5 bar) and shorter times (30 min to 4 h) than the literature precedents (operating up to 70 bar and 300 °C, see Table S4 in the Supplementary Materials for a selection of recent contributions).

## Figures and Tables

**Table 1:** Magnetic parameters for supported metal NPs.

Sample	H_c_ (Oe)	M_r_^MNPs^ (emu.g^−1^)	M_s_^MNPs^ (emu.g^−1^)
NiNP@MgAl_2_O_4_	775	25.2	63.7
NiNP@TiO_2_	947	8.3	29.1
CoNP@TiO_2_	982	13.7	72.0
NiCoNP@MgAl_2_O_4_	181	6.7	156.2
NiCoNP@TiO_2_	511	2.7	52.0

**Table 2:**
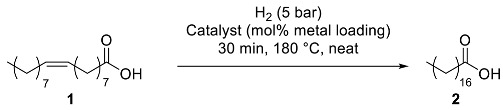
Catalyst screening for the catalyzed hydrogenation of oleic acid using **NiNP@TiO_2_**, **CoNP@TiO_2_**, **NiCoNP@TiO_2_**, **NiNP@MgAl_2_O_4_**, **CoNP@MgAl_2_O_4_**, and **NiCoNP@MgAl_2_O_4_** composite materials ^a^.

Entry	Catalyst	Metal Loading (mol%)	Conv. (%) ^b^
1	**CoNP@TiO_2_**	1	10
2	**NiNP@TiO_2_**	1	65
3	**NiCoNP@TiO_2_**	1	31
4	**CoNP@MgAl_2_O_4_**	1	12
5	**NiNP@MgAl_2_O_4_**	1	96
6	**NiCoNP@MgAl_2_O_4_**	1 ^c^	93
7	**NiCoNP@MgAl_2_O_4_**	0.6 ^d^	91
8	**NiNP@MgAl_2_O_4_**	0.6	35

^a^ Reaction conditions: 1 mmol of oleic acid (**1**), metal content determined by ICP-AES. ^b^ Determined by ^1^H NMR using 4-methylanisole as internal standard; only stearic acid was observed. ^c^ 0.5 mol% Ni and 0.5 mol% Co. ^d^ 0.3 mol% Ni and 0.3 mol% Co.

**Table 3:**
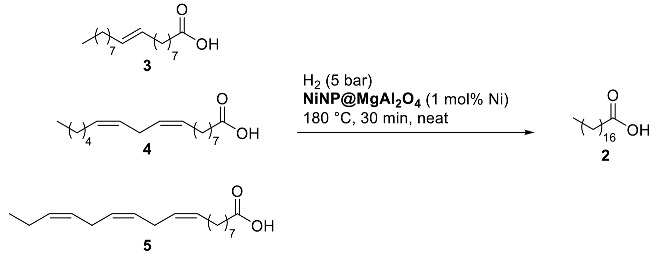
**NiNP@MgAl_2_O_4_**-catalyzed hydrogenation of unsaturated fatty acids, including industrial waste ^a^.

Entry	Substrate	Conv. (%) ^b^	Selectivity (%) ^b^
1	**3**	72	100 ^c^
2	**4**	97	70/30 ^d^
3 ^e^	**5**	86	100 ^d^
4 ^f^	**4**	>99	100 ^c^
5 ^e,f^	**5**	>99	100 ^c^
6 ^f^	Agri-food waste ^g^	>99	96 ^c^ (4) ^h^

^a^ Reaction conditions: 1 mmol of fatty acid, 0.01 mmol of Ni (determined by ICP-AES) unless otherwise stated. ^b^ Determined by ^1^H NMR using 4-methylanisole as internal standard. ^c^ Only stearic acid was observed as a product. ^d^ Unsaturated fatty acids/stearic acid ratio. ^e^ The commercial linolenic acid used contained 20% of linoleic acid as an impurity. ^f^ Reaction time of 4 h using a 2 mol% Ni catalyst loading. ^g^ Composition of saponified raw material: 61% oleic acid, 12% linoleic acid, 3% linolenic acid, 20% stearic acid, and 4% palmitic acid. ^h^ Palmitic acid (4%) from the raw agri-food waste.

## Data Availability

Not applicable.

## Supplementary Materials

The following supporting information can be downloaded at: https://www.mdpi.com/article/10.3390/nano13091435/s1, General experimental information; Figure S1: TEM image of **NiNP@MgAl_2_O_4_** (a), MgAl_2_O_4_ (b), and NiNP (c) after extraction with glycerol size histogram of the isolated NiNP; Figure S2: TEM image of **NiNP@TiO_2_** (a), TiO_2_ (b), and NiNP (c) after extraction with glycerol size histogram of the isolated NiNP; Figure S3: TEM image of **CoNP@MgAl_2_O_4_** (a), MgAl_2_O_4_ (b), and CoNP (c) after extraction with glycerol size histogram of the isolated CoNP; Figure S4: TEM image of **CoNP@TiO_2_** (a), TiO_2_ (b), and CoNP (c) after extraction with glycerol size histogram of the isolated CoNP; Figure S5: TEM images of **NiCoNP@MgAl_2_O_4_** (a), MgAl_2_O_4_ support after extraction of NiCoNP in glycerol (b), and glycerol phase (c); Figure S6: TEM images of **NiCoNP@TiO_2_** (a), TiO_2_ support after extraction of NiCoNP in glycerol (b), and extracted NiCoNP (c). Size distribution (d) for extracted NiCoNP; Figure S7: HRTEM image for **NiCoNP@TiO_2_**; Figure S8: PXRD diffractogram of **NiNPMgAl_2_O_4_**. The (*h k l*) crystallographic planes correspond to MgAl_2_O_4_ spinel structure; Figure S9: (a) Full PXRD diffractogram of NiNP@TiO_2_. (b) Magnification of the 33–56° region. The black (*h k l*) crystallographic planes correspond to the anatase TiO_2_, while the blue (*h k l*) ones correspond to fcc nickel(0). The * symbol corresponds to rutile TiO_2_; Figure S10: PXRD diffractogram of **NiNPMgAl_2_O_4_** (left) and **CoNPMgAl_2_O_4_** (right). The (*h k l*) crystallographic planes correspond to MgAl_2_O_4_ spinel structure; Figure S11: (a) Full PXRD diffractogram of **CoNP@TiO_2_**. (b) Magnification of the 33–56° region. The black (*h k l*) crystallographic planes correspond to the anatase TiO_2_, while the orange (*h k l*) crystallographic planes correspond to fcc cobalt (0). The * symbol corresponds to rutile TiO_2_; Figure S12: (a) Full PXRD diffractogram of **NiCoNP@TiO_2_**. (b) Magnification of the 33–52° region. The black (*h k l*) crystallographic planes correspond to the anatase TiO_2_. The * symbol corresponds to rutile TiO_2_; Figure S13: PXRD diffractogram of **NiCoNP@MgAl_2_O_4_**. The black (*h k l*) crystallographic planes correspond to MgAl_2_O_4_ spinel structure; Figure S14: Stacked DRIFT spectra of **NiNP@TiO_2_** (black), **CoNP@TiO_2_** (blue), **NiCoNP@TiO_2_** (green) with substraction of TiO_2_ signals and quinidine (red); Figure S15: Stacked DRIFT spectra of **NiNP@MgAl_2_O_4_** (black), **CoNP@MgAl_2_O_4_** (blue) and **NiCoNP@MgAl_2_O_4_** (green) without substraction of MgAl_2_O_4_ signals; Figure S16: Nickel L_2,3_ XAS spectra of **NiNP@TiO_2_** (orange), **NiNP@MgAl_2_O_4_** (blue), **NiCoNP@TiO_2_** (green), and **NiCoNP@MgAl_2_O_4_** (red) with references of NiO (black), Ni(OH)_2_ (black dotted line) and Ni(0) (grey); Figure S17: Cobalt L_2,3_ XAS spectra of **NiCoNP@MgAl_2_O_4_** (red), **NiCoNP@TiO_2_** (green), Co(0) reference (grey), and an atomic multiplet simulation of Co(II) (black dotted line); Figure S18: XPS analyses: Ni 2p XPS core level spectra of Ni metal, NiO and Ni(OH)_2_ and corresponding envelopes from the Ni 2p_3/2_ fit (left); Co 2p XPS core level spectra of Co metal, Co_3_O_4_, CoO and Co(OH)_2_ and corresponding envelopes from the Co 2p_3/2_ fit (right); Table S1: Elemental and ICP-AES analyses; Table S2: XPS quantification (in at.%) of MgAl_2_O_4_, **NiNP@MgAl_2_O_4_** and **NiCoNP@MgAl_2_O_4_**; Table S3: Catalyst screening for the catalyzed hydrogenation of oleic acid using **NiNP@TiO_2_**, **NiCoNP@TiO_2_**, **NiNP@MgAl_2_O_4_** and **NiCoNP@MgAl_2_O_4_** composite materials; Table S4: Literature data on the catalyzed hydrogenation of oleic acid. References [49,50,51,52] are cited in the Supplementary Materials.
