# Supplementary material for: Novel Catalyst Composites of Ni- and Co-Based Nanoparticles Supported on Inorganic Oxides for Fatty Acid Hydrogenations
Source: Nanomaterials (Basel). 2023 Apr 22;13(9):1435. doi: 10.3390/nano13091435 (PMC10180328; doi:10.3390/nano13091435)
Supplement: Supplementary file 1 [file nanomaterials-13-01435-s001.zip › nanomaterials-2354546-supplementary.pdf]

Supplementary Materials

Contents:

1. General experimental section

2. Figures

3. Tables

1. General experimental section

All reagents were acquired from commercial suppliers and used as received: bis(1,5-cyclooctadiene)nickel(0) (Strem, 98%), tris(trans-1,2-bis(4-*tert*-butylphenyl)ethene)nickel(0) (Strem, 97%), dicobalt octacarbonyl (Sigma-Aldrich, ≥90%), quinidine (Acros Organics, 98%), halloysite nanoclay (Sigma-Aldrich), oleic acid (Sigma-Aldrich, 95%), linoleic acid (Fluorochem, 94%), linolenic acid (Fluorochem, 70%), elaidic acid (Acros Organics, 98%), 1,4-dimethoxybenzene (Aldrich, 99%). Halloysites and quinidine were dried under vacuum at 80 °C overnight prior to use. Glycerol was degasified and dried under vacuum at 120 °C overnight prior to use. NMR spectra of reaction crudes were recorded on a Bruker Advance 300 spectrometer at 293 K (300 MHz for ^{1}H NMR and 75.4 MHz for ^{13}C NMR) at the NMR service of "Institut de Chimie de Toulouse" and chemical shifts were calibrated relative to residual solvent peak.IR spectra were recorded in the range of 4000-400 cm^{-1} on a ThermoNicolet 6700 FT-IR Spectrometer at the infrared and Raman service of "Institut de Chimie de Toulouse". X-ray powder diffraction analyses were recorded at room temperature on a PANalytical X'Pert Pro MPD (θ-θ) diffractometer or on a MiniFlex Rigaku (θ-2θ) diffractometer at the XRD service of the Coordination Chemistry Laboratory of Toulouse, using Cu $K_{\alpha1,\alpha2}$ radiation (λ= 1.54059, 1.54442 Å). X-Ray diffraction patterns were collected at room temperature on a PANalytical X'Pert MPD Pro (θ-θ) diffractometer using Cu $K\alpha1$,$K\alpha2$ radiation. GC analyses were performed on a GC Perkin Elmer Clarus 500 with a flame ionization detector (FID) using a SGE BPX5 column (30 m x 0.32 mm x 0.25 mm) composed of 5% phenylmethylsiloxane. The injector temperature was 250 °C and the flow was 2 mL/min. The temperature programme was 45 °C for 2 min, 20 °C/min to 300 °C, hold for 5 min. Elemental and ICP-AES analyses were carried out at the elemental analysis service of the Coordination Chemistry Laboratory of Toulouse using a Perkin Elmer 2400 series II analyzer and an iCAP 6300 ICP Spectrometer. Metallic content was determined after sample digestion with aqua regia followed by a dilution of the mixture with HCl 1% (v/v) by inductively coupled plasma atomic emission spectroscopy (ICP-AES) using a Thermo Scientific ICAP 6300 instrument. Magnetic data were collected with a Quantum Design MPMS-X SQUID magnetometer working in the range 2–400 K with the magnetic field up to X Tesla at the magnetism service of the Coordination Chemistry Laboratory of Toulouse. TEM images of nanoparticles both in solid state and dispersed in glycerol were obtained from transmission electron microscopes JEOL JEM 1400 running at 120 kV. HR-TEM from JEOL JEM 2100F running at 200 kV equipped with X PGT (detection of light elements, resolution 135 eV) at the Centre Raimond Castaing of Toulouse. The nanoparticles size, distribution and average diameter were determined from TEM images with Image-J software associated to a Microsoft Excel macro developed by Christian Pradel. X-ray photoelectron spectroscopy (XPS) experiments were carried

out at the Institute of Analytical Sciences and Physico-Chemistry for Environment and Materials (IPREM) using a Thermo Scientific K-alpha spectrometer, with a monochromatized Al Kα radiation (hν = 1486.6 eV). As prepared powder samples were grinded in an agate mortar for 3 min in an Ar filled glove box connected to the spectrometer (H_2O and O_2< 1ppm), deposited on a sample holder using a carbon conductive tape, then immediately transferred in the spectrometer. Measurements were performed at about 10^{-7} Pa with an X-ray power of 72 W (12 kV, 6 mA), corresponding to a 400 μm beam diameter. A dual beam charge neutralization system (low energy electrons and Ar^+ ions flood) was used to avoid charging effect during acquisition. Survey and core level spectra were recorded using 200 or 20 eV as pass energy and 1 or 0.1 eV step, respectively. CasaXPS software was used for peaks fitting. Energy calibration was performed from the C-C, C-H peak at 285.0 eV while non-linear Shirley-type background was used with 70% Gaussian - 30% Lorentzian Voigt peak shapes. Quantification were performed using the Scofield cross-sections database. To fit the Ni $2p_{3/2}$ core level spectra, envelopes corresponding to the Ni $2p_{3/2}$ core level spectra of Ni metal, NiO and $Ni(OH)_2$ reference compounds were used. Note that the Shirley-type background used for the Ni $2p_{3/2}$ fit was adjusted at high energy with a slight offset to improve the fitting corresponding of the satellite peaks area, as previously proposed by Biesinger et al.[1] To fit the Co $2p_{3/2}$ core level spectra, envelopes corresponding to the Co $2p_{3/2}$ core level spectra of Co metal, Co_3O_4, CoO, $Co(OH)_2$ and reference compounds were used. Both Ni and Co reference compounds were recorded in the same analysis conditions while their corresponding maximum peak positions are in good agreement with the literature [1-3]. X ray absorption spectroscopy (XAS) was performed on TEMPO beamline at the Synchrotron SOLEIL [4]. XAS was carried out in partial electron yield by measuring secondary photoelectrons at fixed kinetic energy as a function of the photon energy. Secondary photoelectrons were measured using a hemispherical electron analyzer MBS-A1 equipped with a fast delay line detector. Experimental Nickel and Cobalt absorption spectra were measured at $L_{2,3}$ absorption edge.

1. Biesinger, M.C.; Payne, B.P.; Lau, L.W.M.; Gerson, A.; Smart, R.S.C. X-ray photoelectron spectroscopic chemical state quantification of mixed nickel metal, oxide and hydroxide systems. *Surf. Interface Anal.* **2009**, *41*, 324–332. doi:10.1002/sia.3026.
2. Biesinger, M.C.; Lau, L.W.; Gerson, A.R.; Smart, R.S.C. The role of the Auger parameter in XPS studies of nickel metal, halides and oxides. *Phys. Chem. Chem. Phys.* **2012**, *14*, 2434–2442.
3. Biesinger, M.C.; Payne, B.P.; Grosvenor, A.P.; Lau, L.W.M.; Gerson, A.R.; Smart, R.S.C. Resolving surface chemical states in XPS analysis of first row transition metals, oxides and hydroxides: Cr, Mn, Fe, Co and Ni. *Appl. Surf. Sci.* **2011**, *257*, 2717–2730, doi:10.1016/j.apsusc.2010.10.051.
4. Polack, F.; Silly, M.; Chauvet, C.; Lagarde, B.; Bergeard, N.; Izquierdo, M.; Chubar, O.; Krizmancic, D.; Ribbens, M.; Duval, J.P.; et al. TEMPO: a New Insertion Device Beamline at SOLEIL for Time Resolved Photoelectron Spectroscopy Experiments on Solids and Interfaces. *AIP Conf. Proc.* **2010**, *1234*, 185–188, doi:10.1063/1.3463169.

2. Figures

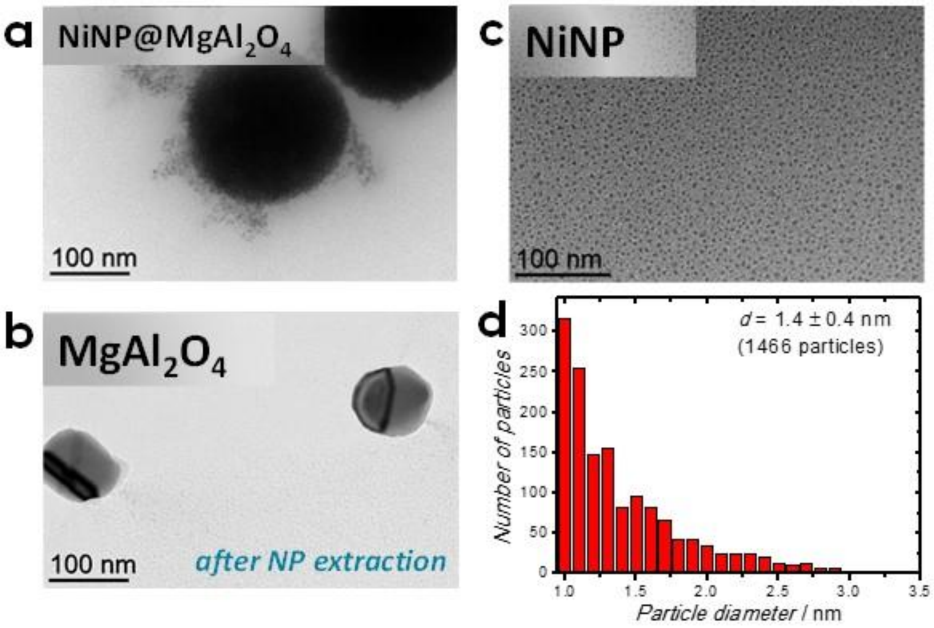

Figure S1. TEM image of **NiNP@MgAl$_2$O$_4$** (a), MgAl$_2$O$_4$ (b), and NiNP (c) after extraction with glycerol size histogram of the isolated NiNP.

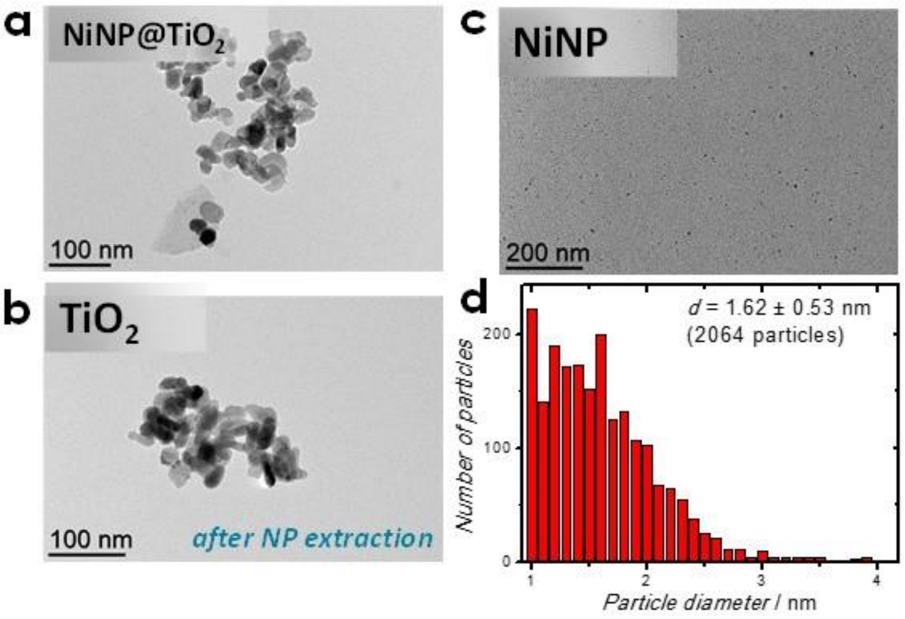

Figure S2. TEM image of **NiNP@TiO$_2$** (a), TiO$_2$ (b), and NiNP (c) after extraction with glycerol size histogram of the isolated NiNP.

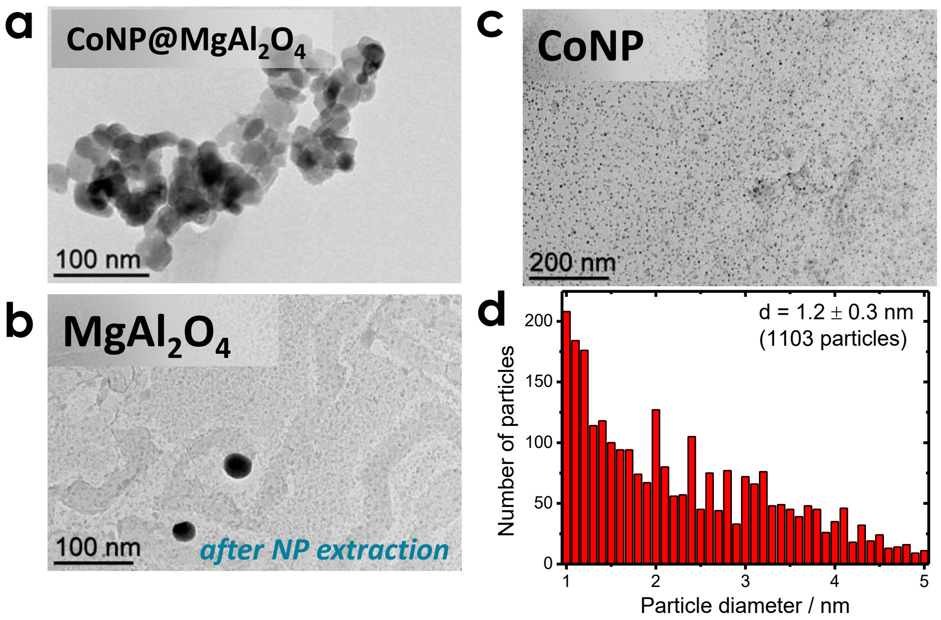

Figure S3. TEM image of **CoNP@MgAl$_2$O$_4$** (a), MgAl$_2$O$_4$ (b), and CoNP (c) after extraction with glycerol size histogram of the isolated CoNP.

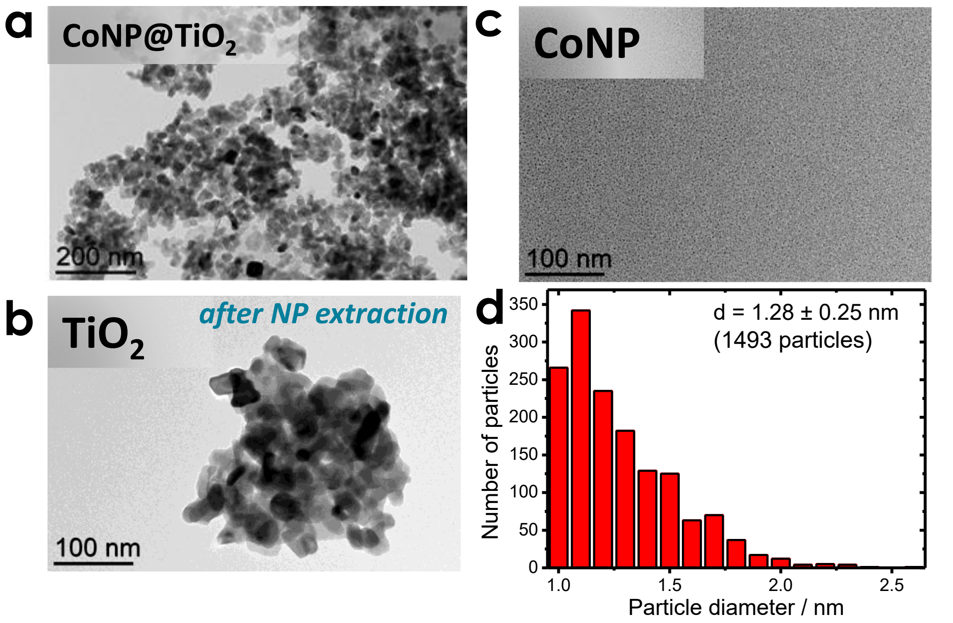

Figure S4. TEM image of **CoNP@TiO$_2$** (a), TiO$_2$ (b), and CoNP (c) after extraction with glycerol size histogram of the isolated CoNP.

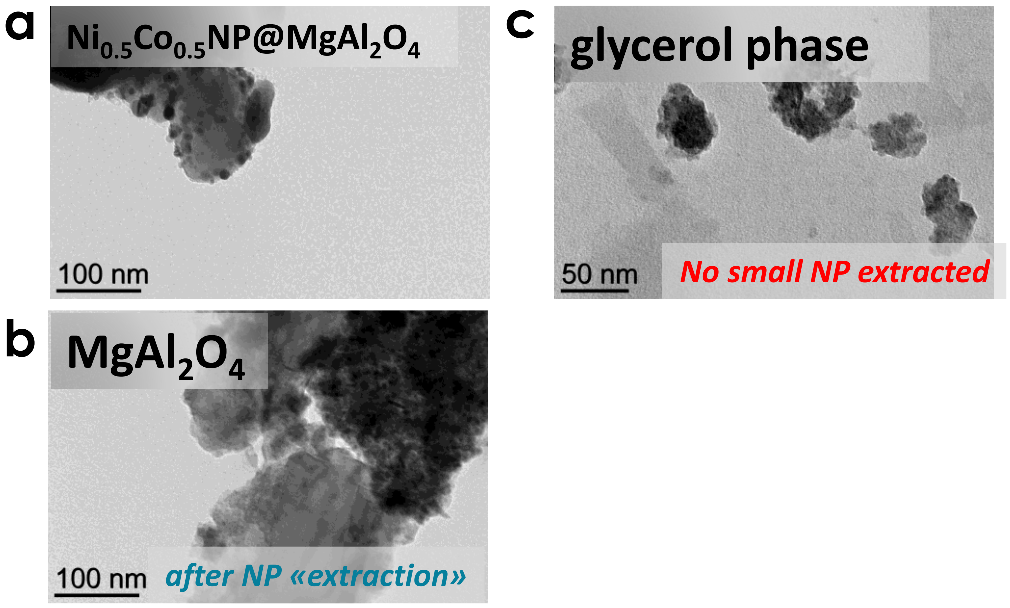

Figure S5. TEM images of **NiCoNP@$MgAl_2O_4$** (a), $MgAl_2O_4$ support after extraction of NiCoNP in glycerol (b), and glycerol phase (c).

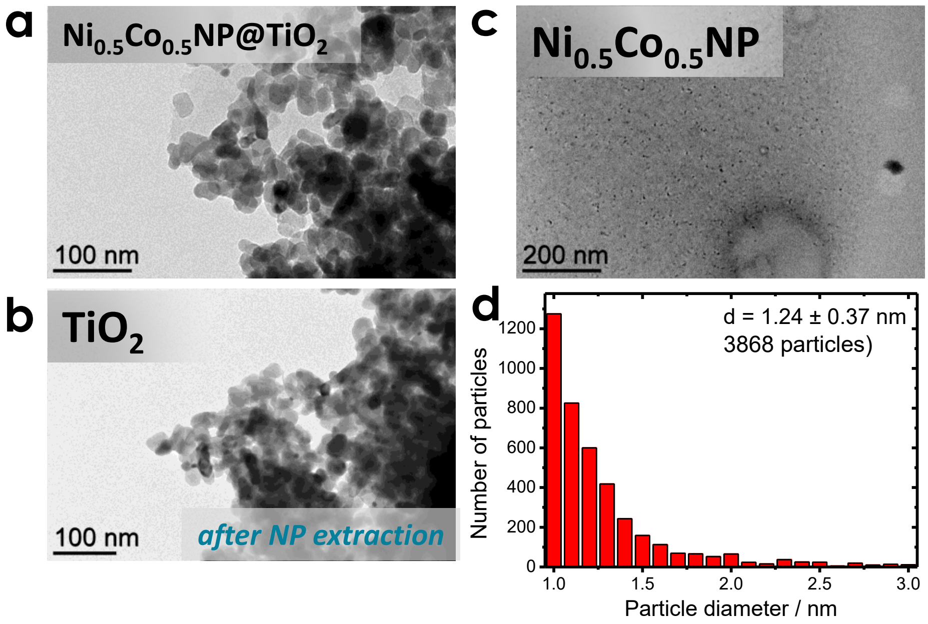

Figure S6. TEM images of **NiCoNP@TiO_2** (a), TiO_2 support after extraction of NiCoNP in glycerol (b), and extracted NiCoNP (c). Size distribution (d) for extracted NiCoNP.

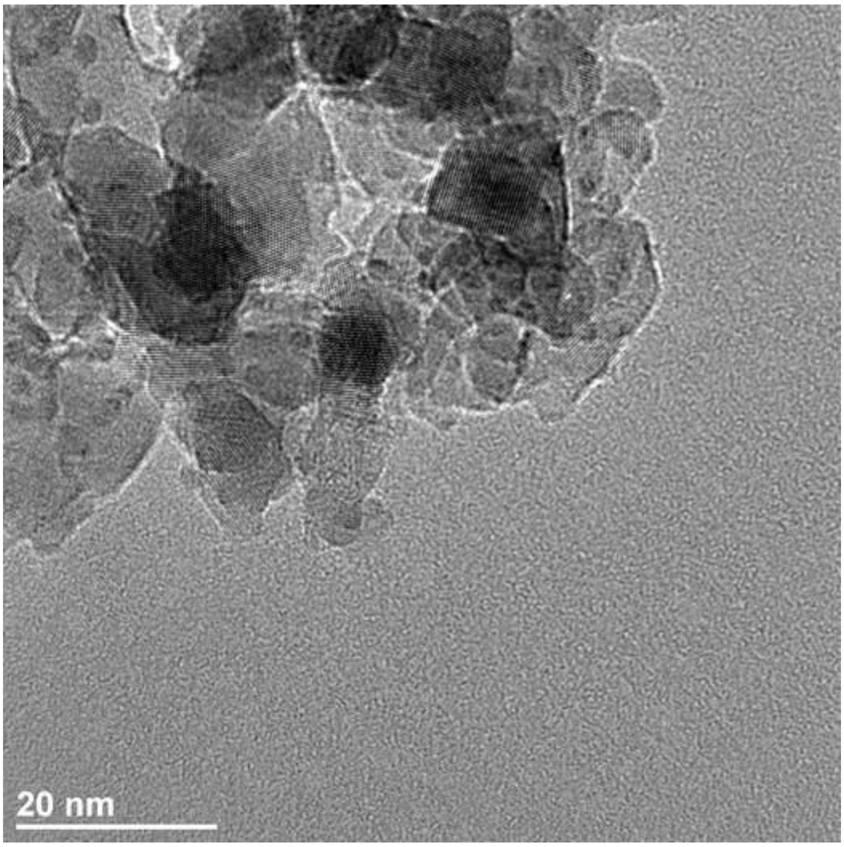

Figure S7. HRTEM image for **NiCoNP@TiO$_2$**.

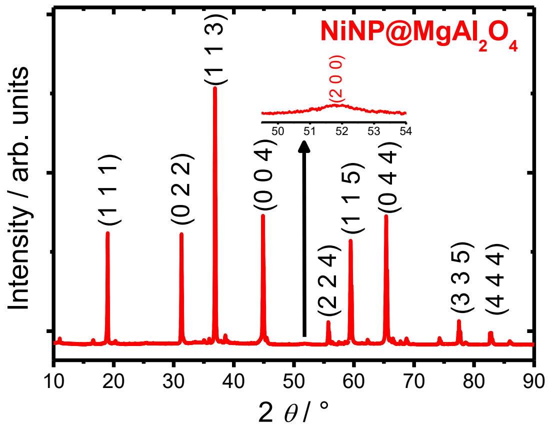

Figure S8. PXRD diffractogram of **NiNPMgAl2O4**. The *(h k l)* crystallographic planes correspond to MgAl2O4 spinel structure.

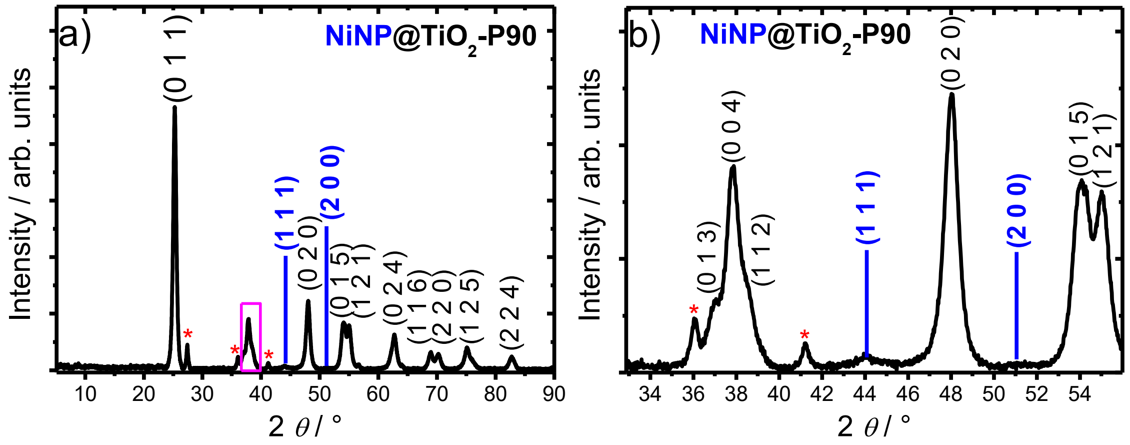

Figure S9. (a) Full PXRD diffractogram of **NiNP@TiO2**. (b) Magnification of the 33-56° region. The black *(h k l)* crystallographic planes correspond to the anatase TiO2, while the blue *(h k l)* ones correspond to fcc nickel(0). The * symbol corresponds to rutile TiO2.

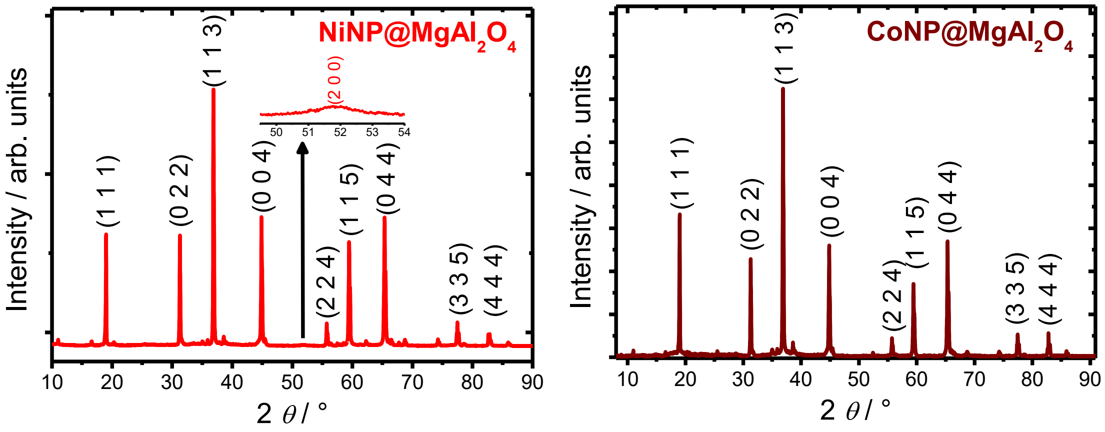

Figure S10. PXRD diffractogram of **NiNPMgAl2O4** (left) and **CoNPMgAl2O4** (right). The *(h k l)* crystallographic planes correspond to MgAl2O4 spinel structure.

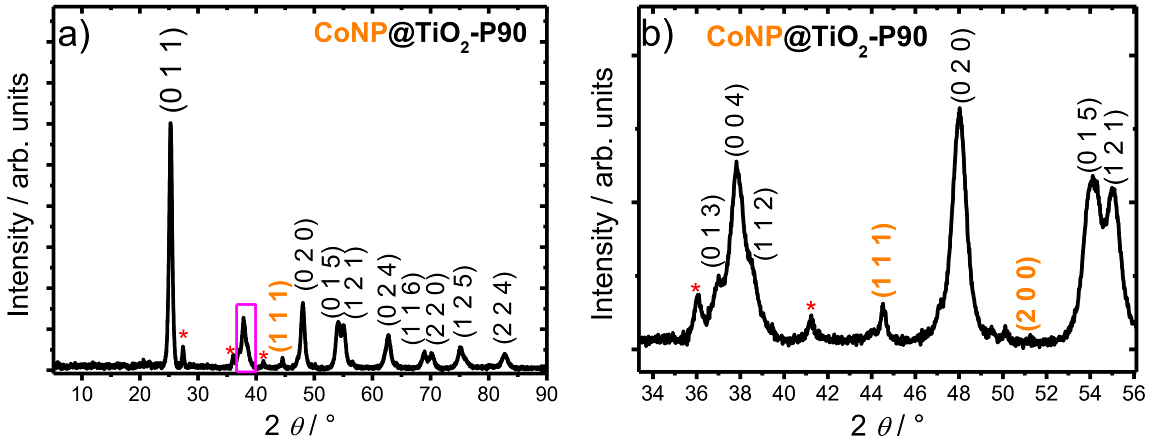

Figure S11. (a) Full PXRD diffractogram of **CoNP@TiO2**. (b) Magnification of the 33-56° region. The black *(h k l)* crystallographic planes correspond to the anatase TiO_2, while the orange *(h k l)* crystallographic planes correspond to fcc cobalt (0). The * symbol corresponds to rutile TiO_2.

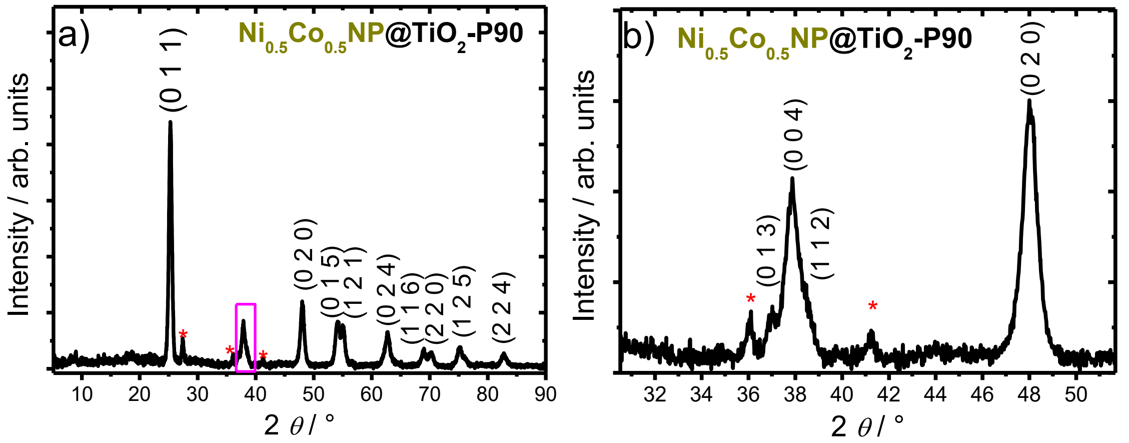

Figure S12. (a) Full PXRD diffractogram of **NiCoNP@TiO2**. (b) Magnification of the 33-52° region. The black *(h k l)* crystallographic planes correspond to the anatase TiO_2. The * symbol corresponds to rutile TiO_2.

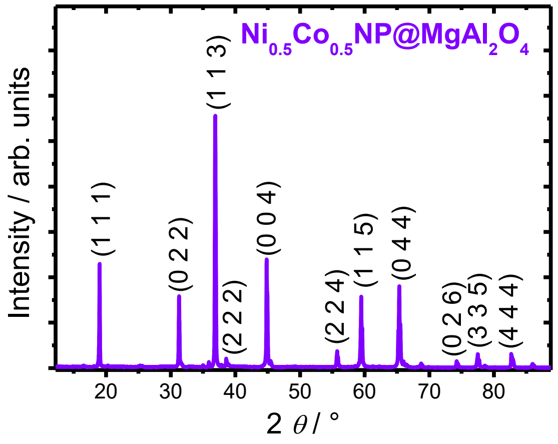

Figure S13. PXRD diffractogram of **NiCoNP@MgAl2O4**. The black *(h k l)* crystallographic planes correspond to $MgAl_2O_4$ spinel structure.

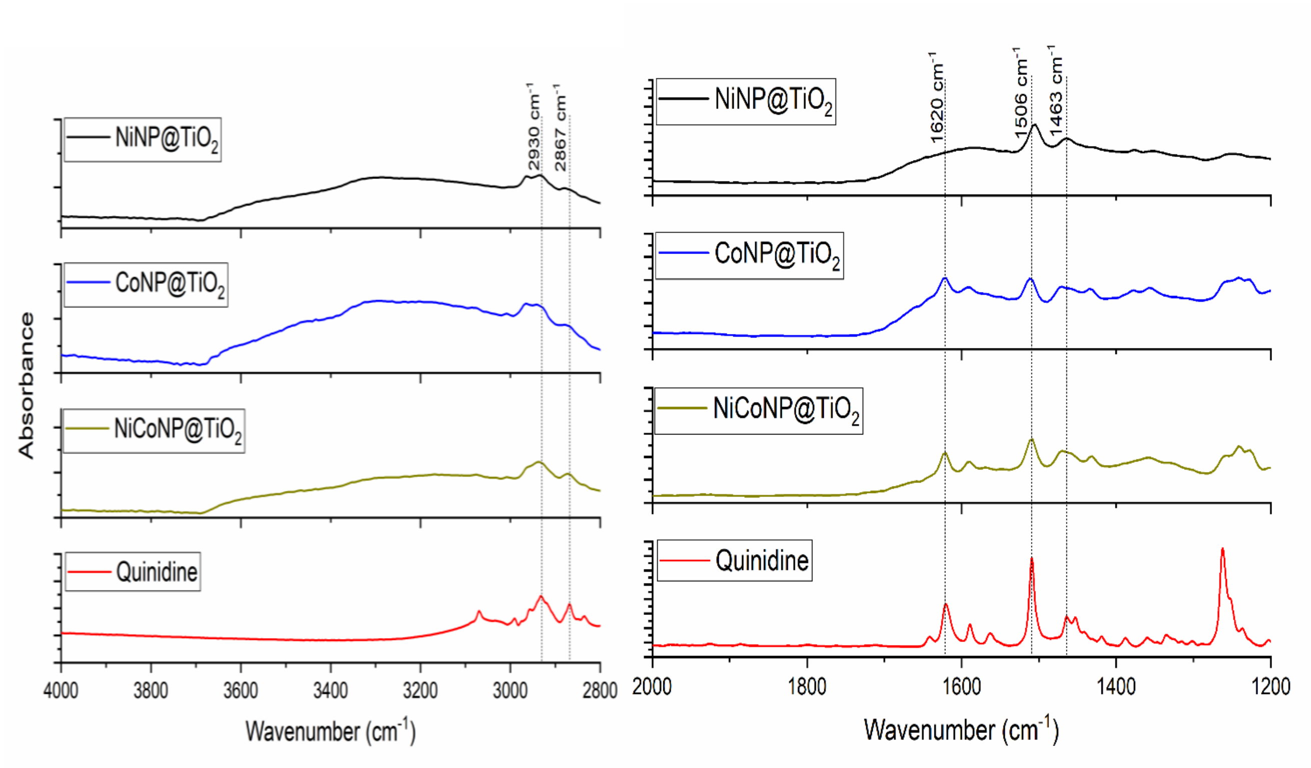

Figure S14. Stacked DRIFT spectra of **NiNP@TiO₂** (black), **CoNP@TiO₂** (blue), **NiCoNP@TiO₂** (green) with substraction of TiO₂ signals and quinidine (red).

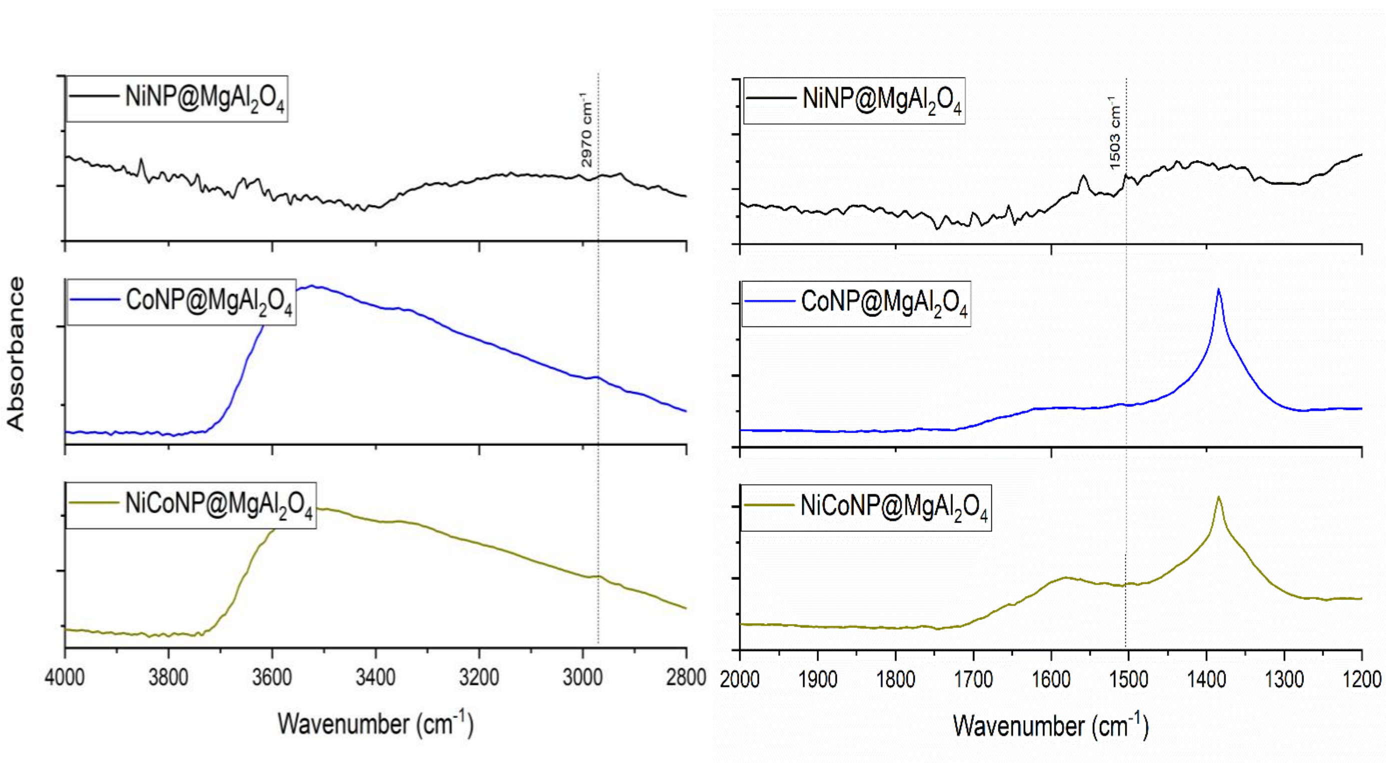

Figure S15. Stacked DRIFT spectra of **NiNP@MgAl₂O₄** (black), **CoNP@MgAl₂O₄** (blue) and **NiCoNP@MgAl₂O₄** (green) without substraction of MgAl₂O₄ signals.

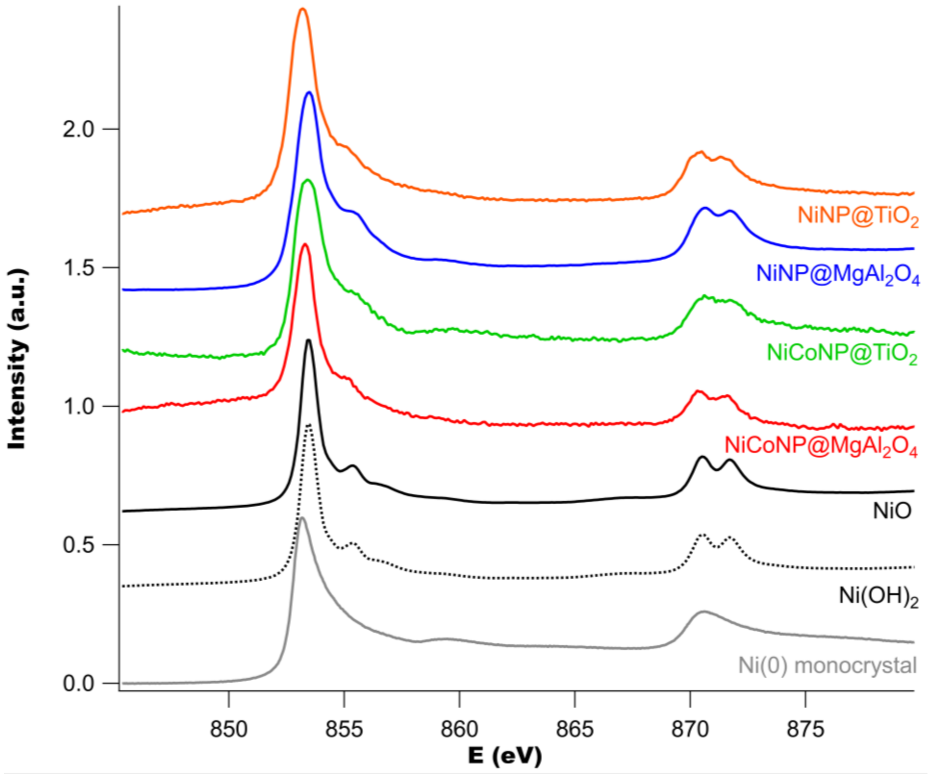

Figure S16. Nickel $L_{2,3}$ XAS spectra of **NiNP@TiO$_2$** (orange), **NiNP@MgAl$_2$O$_4$** (blue), **NiCoNP@TiO$_2$** (green), and **Ni-CoNP@MgAl$_2$O$_4$** (red) with references of NiO (black), Ni(OH)$_2$ (black dotted line) and Ni(0) (grey).

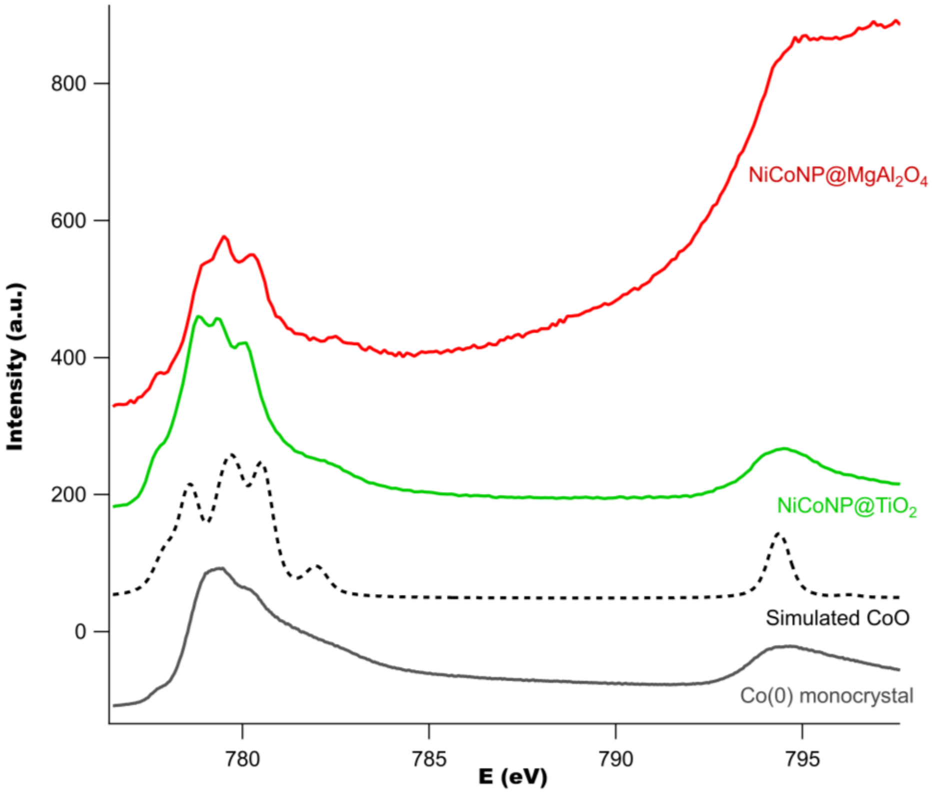

Figure S17. Cobalt $L_{2,3}$ XAS spectra of **NiCoNP@MgAl$_2$O$_4$** (red), **NiCoNP@TiO$_2$** (green), Co(0) reference (grey), and an atomic multiplet simulation of Co(II) (black dotted line).

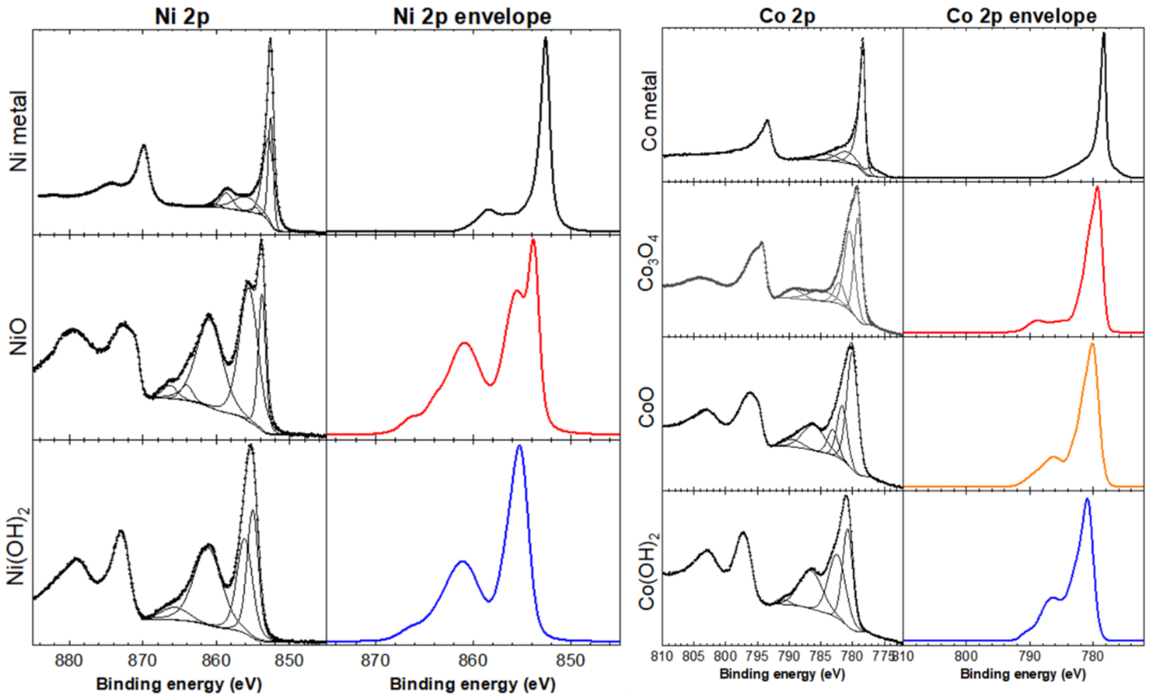

Figure S18. XPS analyses: Ni 2p XPS core level spectra of Ni metal, NiO and $Ni(OH)_2$ and corresponding envelopes from the Ni $2p_{3/2}$ fit (left); Co 2p XPS core level spectra of Co metal, Co_3O_4, CoO and $Co(OH)_2$ and corresponding envelopes from the Co $2p_{3/2}$ fit (right).

3. Tables

Table S1. Elemental and ICP-AES analyses.

Sample	ICP-AES		Elemental Analysis	
	Ni (wt%)	Co (wt%)	C (wt%)	N (wt%)
**NiNP@MgAl$_2$O$_4$**	4.63	--	0.60	0.02
**NiNP@TiO$_2$**	4.62	--	2.97	0.22
**CoNP@TiO$_2$**	--	4.56	0.72	0.07
**CoNP@MgAl$_2$O$_4$**	--	4.55	1.03	0.04
**NiCoNP@TiO$_2$**	2.98	1.77	1.24	0.06
**NiCoNP@MgAl$_2$O$_4$**	2.76	2.62	0.78	0.04

Table S2. XPS quantification (in at.%) of MgAl$_2$O$_4$, **NiNP@MgAl$_2$O$_4$** and **NiCoNP@MgAl$_2$O$_4$**.

Elements	Binding energy (eV)	Assignment	At.% MgAl$_2$O$_4$	At.% Ni@MgAl$_2$O$_4$	At.% NiCo@MgAl$_2$O$_4$
C 1s	285<br>286.7<br>289.3	Contamination	14<br>1.5<br>1	20<br>2<br>1.5	6<br>1<br>1
O 1s	531.1<br>532.4	Al$_2$**O$_3$**<br>MgAl$_2$**O$_4$**	36<br>13	Total O:<br>46	30<br>23
Mg 1s	1304	**Mg**Al$_2$O$_4$	3.5	1.5	6
Al 2p	74.5	Mg**Al$_2$**O$_4$/**Al$_2$**O$_3$	31	16.5	31
Ni 2p	852.6 (maximum)<br>854.0 (maximum)<br>856.0 (maximum)	Ni metal<br>NiO<br>Ni(OH)$_2$	-	12.5<br>18%<br>26%<br>56%	1<br>17%<br>53%<br>30%
Co 2p	778.1 (maximum)<br>780.0 (maximum)<br>781.4 (maximum)	Co metal<br>CoO<br>Co(OH)$_2$			1<br>16%<br>40%<br>44%

Table S3. Catalyst screening for the catalyzed hydrogenation of oleic acid using **NiNP@TiO₂**, **NiCoNP@TiO₂**, **NiNP@MgAl₂O₄** and **NiCoNP@MgAl₂O₄** composite materials.[a]

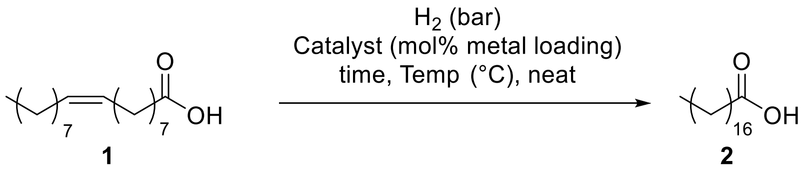

Entry	Catalyst	Metal loading (mol%)	pH_2 (bar)	Temperature (°C)	Time	Conv. (%)[b]
1	**NiNP@TiO₂**	10	10	100	24 h	100
2	**NiNP@TiO₂**	5	10	100	24 h	100
3	**NiNP@TiO₂**	1	5	180	30 min	65
4	**NiNP@TiO₂**	0.3	5	180	30 min	10
5	**CoNP@TiO₂**	1	5	180	30 min	10
6	**NiCoNP@TiO₂**	10	10	100	24 h	38
7	**NiCoNP@TiO₂**	1	5	180	30 min	31
8	**NiCoNP@TiO₂**	0.5	5	180	30 min	19
9	**NiNP@MgAl₂O₄**	5	10	100	24 h	100
10	**NiNP@MgAl₂O₄**	2	5	180	30 min	100
11	**NiNP@MgAl₂O₄**	1	5	180	30 min	96
12	**NiNP@MgAl₂O₄**	0.2	5	180	30 min	32
13	**NiNP@MgAl₂O₄**	0.2	5	100	2 h	13
14	**CoNP@MgAl₂O₄**	1	5	180	30 min	12
15	**NiCoNP@MgAl₂O₄**	10	10	100	24 h	100
16	**NiCoNP@MgAl₂O₄**	5	10	100	24 h	100
17	**NiCoNP@MgAl₂O₄**	1	5	180	30 min	93
18	**NiCoNP@MgAl₂O₄**	0.6	5	180	30 min	91
19[c]	**NiCoNP@MgAl₂O₄**	0.1	5	180	30 min	50
20	**NiCoNP@MgAl₂O₄**	0.1	5	100	2 h	n.d.

[a] Reaction conditions: 1 mmol of oleic acid (**1**), metal content determined by ICP-AES. [b] Determined by ^1H NMR using 4-methylanisole as internal standard; only stearic acid was observed. [c] Reaction carried out using 5 mmol of substrate.

Table S4. Literature data on the catalyzed hydrogenation of oleic acid.

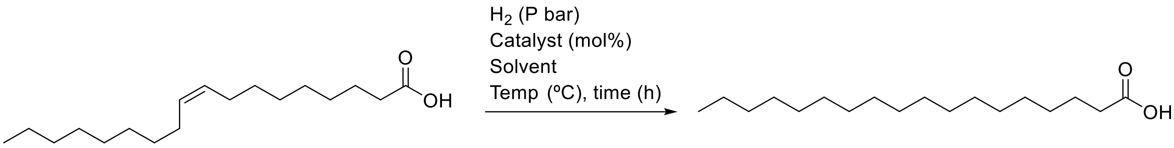

**Catalyst**	**Solvent**	**P_{H_2} (bar)**	**T (°C)**	**Time (h)**	**Yield (%)**	**Reference**
Pd@SiO_2 (0.1 mol%)	MeOH	1	rt	3	>97	*Org. Process. Res. Dev.* **2012**, *16*, 1307
Ag@SiO_2 (0.5 mol%)	EtOH	1	50	5	91	*Mater. Lett.* **2011**, *65*, 1037
η^6-arene Ru diphosphine complex (0.2 mol%)	DME	68 (P_{CO_2} 12)	160	20	(98% methylester)	*Chem. Sci.* **2020**, *11*, 6766
Pt/C (5 wt%)	H_2O	3.5	300	9	75	*Green Chem.* **2014**, *16*, 1507
Pt-Re/C (5 and 4 wt%, respectively)	H_2O	3.5	300	9	65	*Green Chem.* **2014**, *16*, 1507
Fe/mesoporous SiO_2 NP	hexane	30	290	6	83	*J. Catal.* **2014**, *314*, 142
Ni-Mo/Al_2O_3 (5 wt%)	dodecane	49	320	4	61	*Ind. Eng. Chem. Res.* **2017**, *56*, 5547
Ni/$ZnAl_2O_4$ (10 wt%)	Decalin	25	280	6	83	*Catal. Sci. Technol.* **2019**, *9*, 213
Co@Chitosan-700 (2.9 mol%)	H_2O	40	150	18	62 (conversion)	*Sci. Adv.* **2018**, *4*, eaau1248
